# Multifocal Necrotizing Fasciitis of the Bilateral Chest Wall and Contralateral Lower Limb in a Patient With Diabetes: A Case Report

**DOI:** 10.7759/cureus.114487

**Published:** 2026-08-13

**Authors:** Ashfak Hussain, Ibrahim Makhlouf

**Affiliations:** 1 Department of Internal Medicine, Thumbay University Hospital, Ajman, ARE; 2 Department of General Surgery, Thumbay University Hospital, Ajman, ARE

**Keywords:** case report, chest wall, diabetes mellitus, necrotizing fasciitis, polymicrobial infection

## Abstract

Necrotizing fasciitis (NF) is a rapidly progressive, life-threatening infection involving the fascia and subcutaneous tissues. Chest wall NF is rare and typically occurs following thoracic surgery or penetrating trauma. We report a 54-year-old man with type 2 diabetes mellitus who developed bilateral chest wall NF after sustaining a pectoralis major muscle injury while lifting a heavy engine part. Imaging demonstrated bilateral chest wall abscesses with mediastinal extension. During hospitalization, he also developed cellulitis of the contralateral lower limb, which subsequently progressed to NF, with imaging showing diffuse subcutaneous edema of the left lower leg. Wound cultures demonstrated polymicrobial growth, including methicillin-resistant *Staphylococcus aureus*. The patient required multiple surgical debridements, broad-spectrum antimicrobial therapy, strict glycemic control, and coordinated multidisciplinary care, ultimately achieving complete recovery. This case highlights the unusual occurrence of bilateral chest wall NF with mediastinal extension and the rare development of NF in the contralateral lower limb. It emphasizes the importance of early recognition, repeated surgical debridement, and comprehensive whole-body examination in diabetic patients presenting with sepsis, as additional or distant sites of infection may otherwise be overlooked.

## Introduction

Necrotizing fasciitis (NF) is an uncommon but potentially fatal soft-tissue infection characterized by rapidly progressive necrosis of the fascia and subcutaneous tissues. Although the extremities are most frequently affected, chest wall NF is rare and is generally associated with thoracic surgery, penetrating trauma, or direct extension from an adjacent infection. The condition may be caused by *Streptococcus pyogenes*, *Staphylococcus aureus*, or mixed aerobic and anaerobic organisms [1].

Diabetes mellitus is an important predisposing factor for NF because of impaired immune function, microvascular disease, and delayed wound healing. The high prevalence of diabetes mellitus in the United Arab Emirates (UAE) may place a substantial proportion of the population at increased risk, although region-specific epidemiological data regarding NF remain limited. Early recognition, prompt administration of broad-spectrum antimicrobial therapy, and urgent surgical debridement are essential to reduce morbidity and mortality [2].

NF may initially present with nonspecific findings such as fever, tachycardia, pain, swelling, and systemic toxicity, making early diagnosis challenging. Diabetes mellitus is an important predisposing comorbidity and may contribute to rapid progression and poorer outcomes [3]. Chest wall involvement is uncommon and may extend into deeper thoracic structures [1]. In patients with unexplained sepsis, examination of anatomically distant sites should be considered because multifocal, noncontiguous infection, although rare, has been reported [4].

Chest wall NF following blunt, nonpenetrating muscle injury is rarely reported. The present case was unusual because of bilateral chest wall involvement, CT-reported mediastinal extension, and NF of the contralateral left lower limb. This report aims to highlight blunt muscle injury as a possible initiating factor, the potential for anatomically distant involvement, and the importance of comprehensive examination in patients with diabetes presenting with sepsis.

## Case presentation

A 54-year-old man presented to the emergency department on December 7, 2024, because of chest pain that had begun the previous night. The pain initially affected the right upper chest and was associated with chest wall swelling and right shoulder weakness. He stated that he injured the right aspect of his chest while lifting a heavy engine part. He also reported recurrent fever, which had worsened on the night before presentation.

Approximately one week before admission, he had developed painful swelling and erythema of the left lower leg. He attended a local clinic and was prescribed antibiotics and analgesics; however, the symptoms persisted despite treatment. His medical history included type 2 diabetes mellitus diagnosed two years earlier and treated with metformin, as well as a previous left tibial fracture managed with internal fixation.

Physical examination

On examination, the patient looked unwell; he was febrile with a temperature of 39°C, blood pressure of 110/50 mmHg, pulse of 117 beats/minute, and respiratory rate of 23/minute. The skin overlying both the chest was edematous, warm, and tender to touch. The left leg appeared to be swollen with reddish overlying skin, which was tender to touch. Both the chest and left foot demonstrated crepitus and significant induration. The rest of the physical examination was unremarkable. Laboratory investigations are listed in Table 1.

**Table 1:** Summary of baseline laboratory findings, including hematological, inflammatory, metabolic, and arterial blood gas parameters, with corresponding reference ranges

Test	Result with reference range
Hemoglobin	9.5 g/dL (13-16 g/dL)
White blood cells	15,400 cells/mm^3^ (4,000-11,000 cells/mm^3^)
C-reactive protein	115.6 mg/L (<5 mg/L)
Random blood glucose (nonfasting state)	348 mg/dL (70-140 mg/dL)
Glycosylated hemoglobin	7.85% (<5.7%)
pH	7.33 (7.35-7.45)
Bicarbonate	24.4 mEq/L (22-26 mEq/L)
Partial pressure of carbon dioxide	37 mmHg (35-45 mmHg)
Lactic acid	5.2 mmol/L (0.5-2.2 mmol/L)
Partial pressure of oxygen	83 mmHg (80-100 mmHg)
Sodium	132 mEq/L (135-145 mEq/L)
Potassium	3.1 mEq/L (3.5-5.0 mEq/L)
Chloride	99 mEq/L (96-106 mEq/L)
Serum creatinine	1.26 mg/dL (0.7-1.3 mg/dL)
Blood urea nitrogen	53.4 mg/dL (7-20 mg/dL)

Based on the initial admission laboratory values, the Laboratory Risk Indicator for Necrotizing Fasciitis (LRINEC) score was calculated as 6 points. The component scores were as follows: C-reactive protein (CRP) 115.6 mg/L (0 points), white blood cell (WBC) count 15,400 cells/mm^3^ (1 point), hemoglobin 9.5 g/dL (2 points), serum sodium 132 mmol/L (2 points), serum creatinine 1.26 mg/dL (0 points), and serum glucose 348 mg/dL (1 point). This corresponded to an intermediate risk category.

Imaging

**Figure 1:**
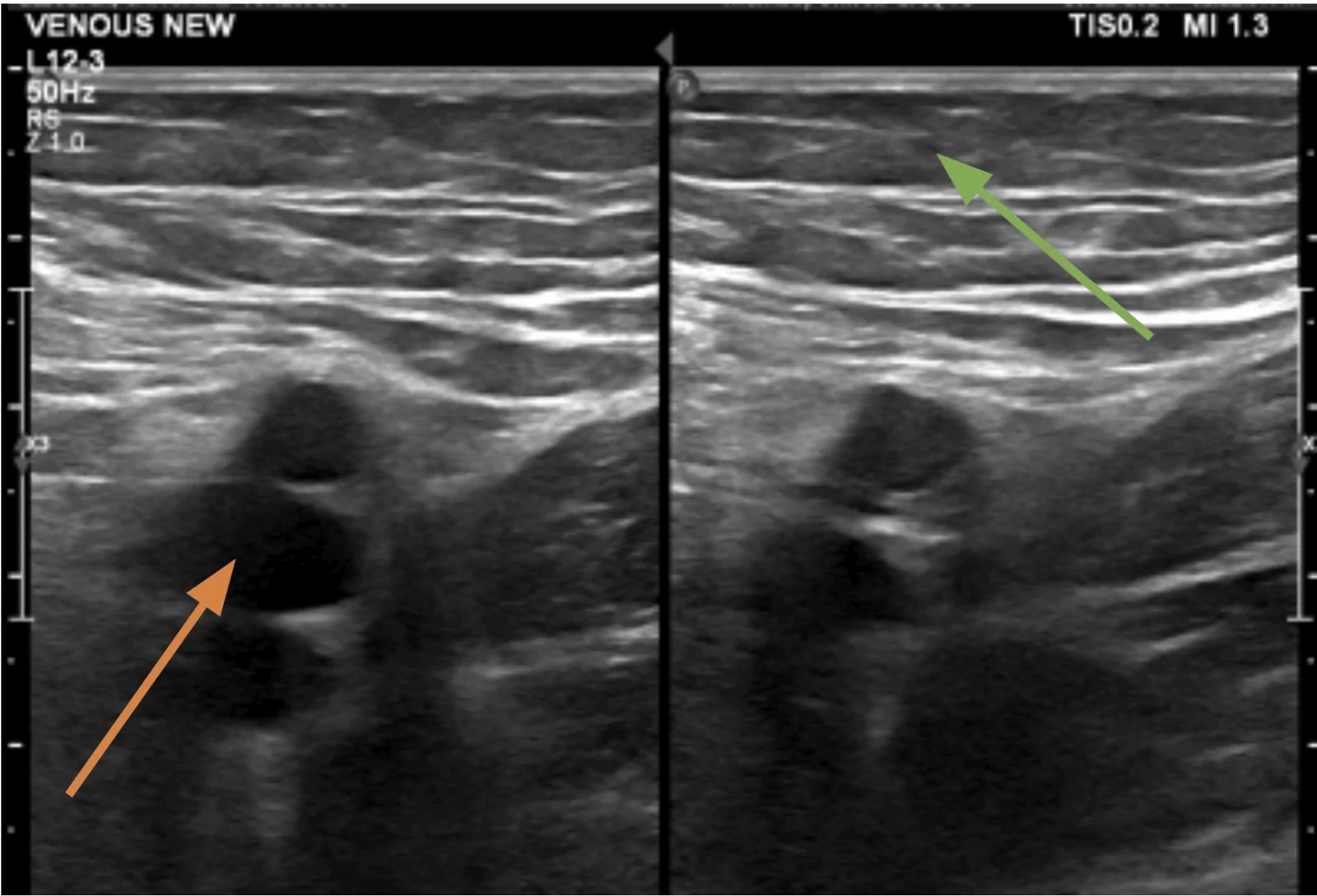
Left lower limb ultrasound The orange arrow points to the fluid streaking at the ankle region, with fluid collection at the tibialis anterior tendon. The green arrow denotes the subcutaneous soft-tissue edema along the left lower leg

An ultrasound performed on the left lower limb depicts diffuse subcutaneous soft-tissue edema along the left lower leg, with associated fluid streaking at the ankle region, with fluid collection at the tibialis anterior tendon (Figure 1).

**Figure 2:**
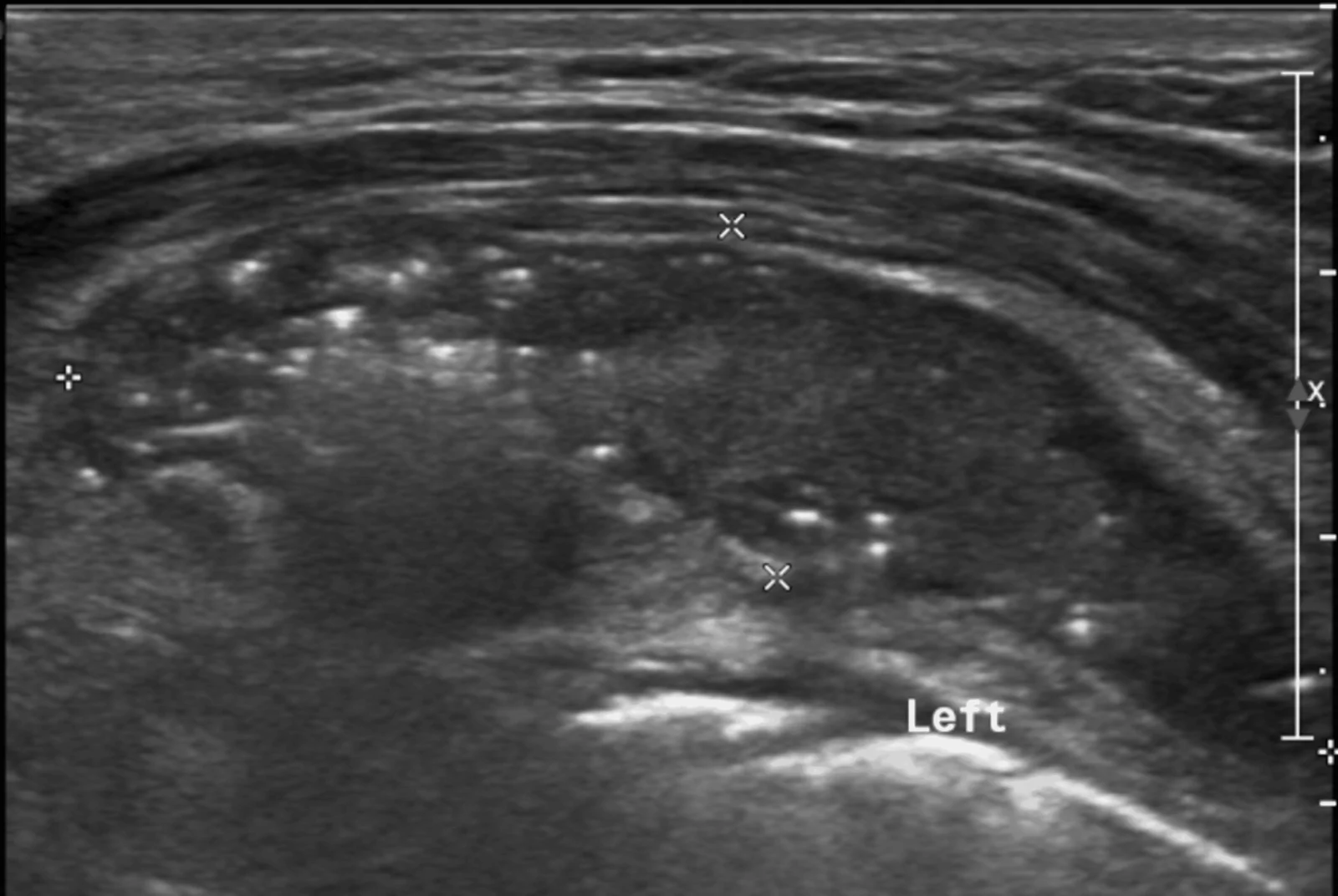
Ultrasound of the left chest mass Ultrasound shows an irregular, hypoechoic soft-tissue mass measuring approximately 4.4 x 2.1 x 2.6 cm, with a hypoechoic extension measuring 2.7 x 2.6 cm traversing the intercostal space into the mediastinum. Multiple internal hyperechoic foci are noted, consistent with intralesional gas, and surrounding soft-tissue planes appear thickened, in keeping with an inflammatory process

**Figure 3:**
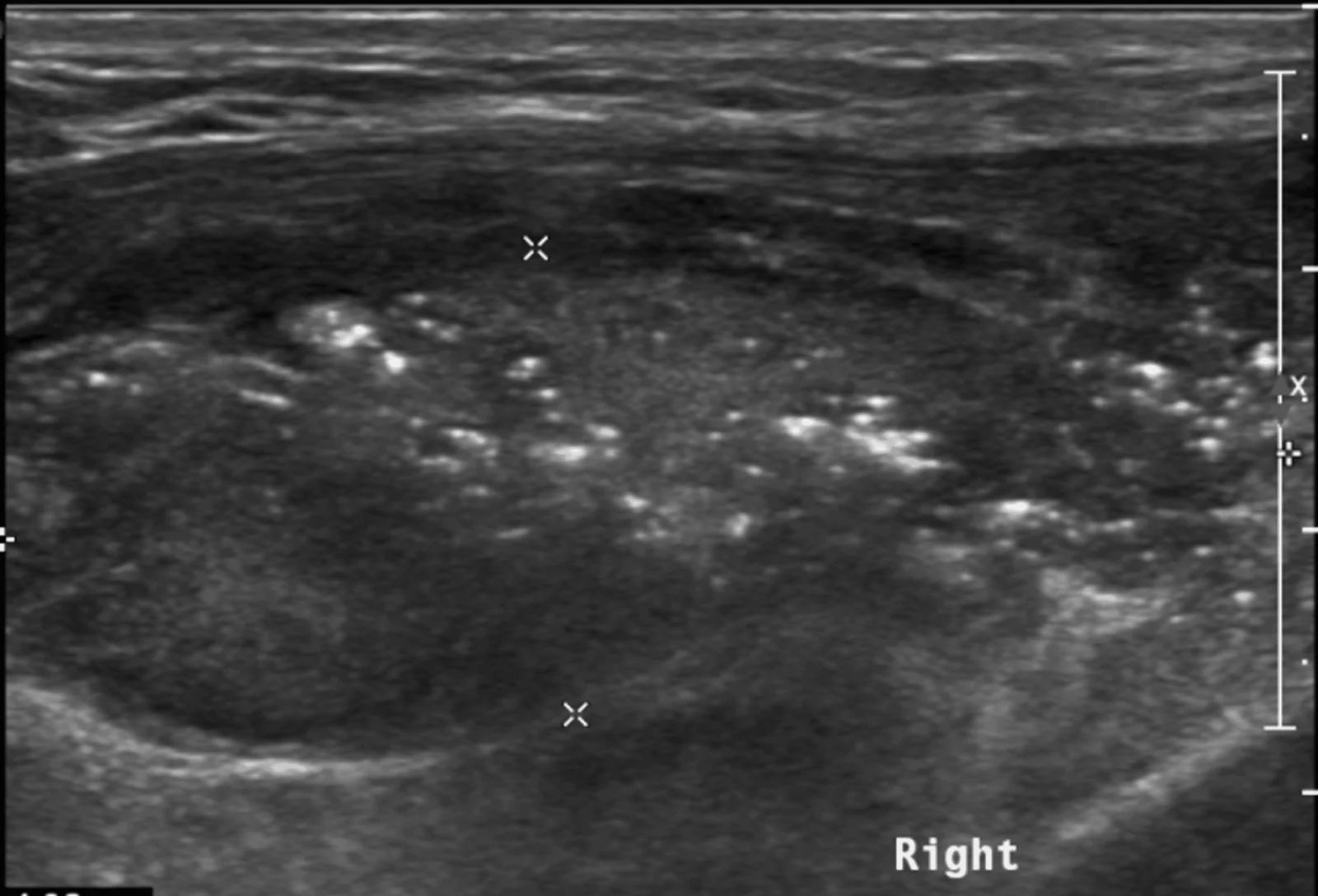
Ultrasound of the right chest mass Multiple internal echogenic foci producing posterior dirty shadowing, compatible with intralesional gas

Ultrasound of the left chest mass showed the presence of a soft-tissue mass with multiple internal hyperechoic foci, which are consistent with intralesional gas. There was also surrounding soft-tissue edema present (Figure 2). Ultrasound of the right chest mass shows similar findings as seen in the left, but the inflammatory process has spread deep into the muscles and axilla, depicting deep fascial and mediastinal involvement (Figure 3).

According to the formal contrast-enhanced CT report of the chest, two anterior chest wall masses were located extending from the intercostal spaces to the anterior mediastinum. The right mass measures 6.8 cm (transverse) × 4.7 cm (craniocaudal) × 4 cm (anteroposterior); it extends laterally between lateral chest wall muscles until the right axilla. The left mass measures 4.4 x 2.1 x 2.6 cm. The masses contained multiple air bubbles, suggesting the presence of necrotizing tissue. However, the original CT images were not available for inclusion in the article.

Intraoperative exploration confirmed extensive necrosis of the chest wall fascia and subcutaneous tissues, with direct extension into the mediastinum. Multiple surgical debridements were required to remove all devitalized tissue and achieve adequate source control.

Treatment

Incision and drainage were performed under general anesthesia via bilateral 4-cm transverse incisions at the level of the sternal angle on December 12, 2024. Surgical exploration of the chest wall demonstrated necrotic subcutaneous tissue and fascia involving the pectoral region. The right pectoralis major muscle was partially dissected as it had undergone severe necrosis. Debridement was continued until viable, bleeding tissue was encountered, followed by drainage and placement of surgical drains.

A separate procedure was done on December 15, which involved an incision made over the medial left ankle for drainage of the abscess. Exploration of the left lower-limb wound included drainage of the collection and excision of nonviable soft tissue. Intravenous vancomycin 1.5 g q12hrs and meropenem 1 g q8hrs were commenced initially on admission. Wound cultures recovered from the chest and left leg demonstrated polymicrobial growth including methicillin-resistant *Staphylococcus aureus* (MRSA), *Escherichia coli*, and Bacteroides and Klebsiella species.

**Figure 4:**
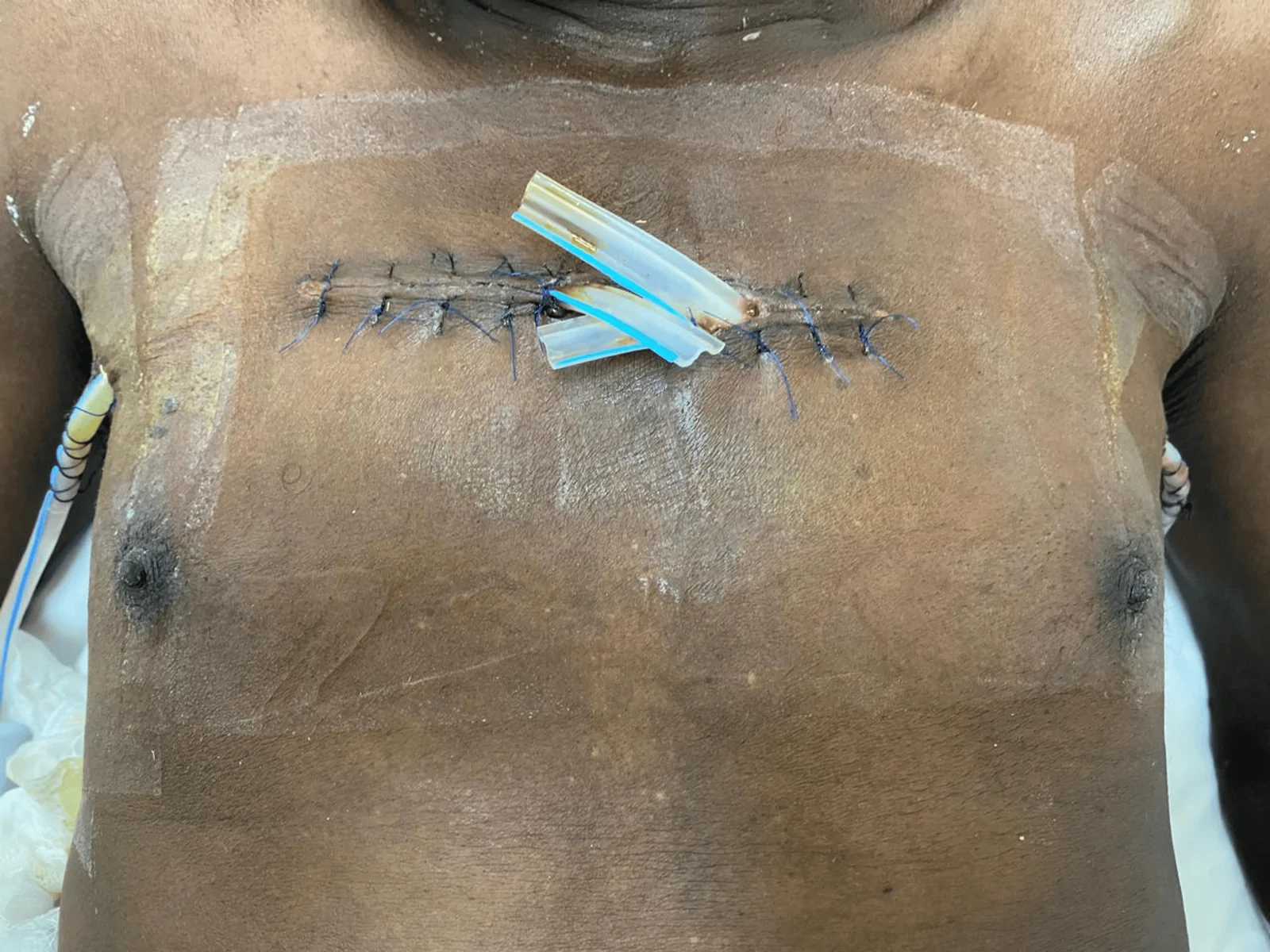
Postoperative appearance of the chest wall following surgical debridement for necrotizing soft-tissue infection. Anterior view demonstrating the transverse upper chest incision and bilateral chest wall drains following debridement

**Figure 5:**
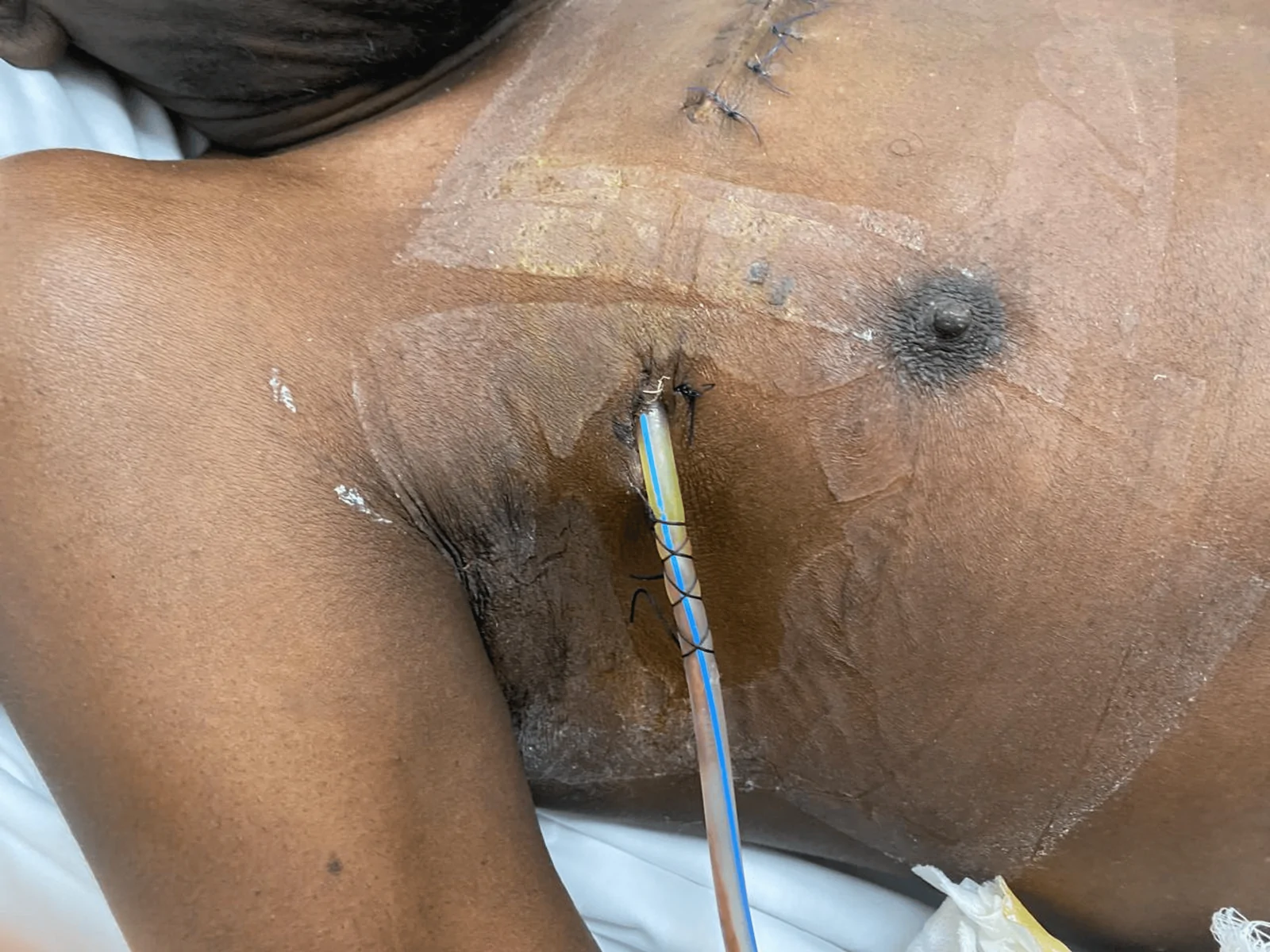
Right lateral chest and axillary region showing a partially closed surgical incision with a drain in situ

**Figure 6:**
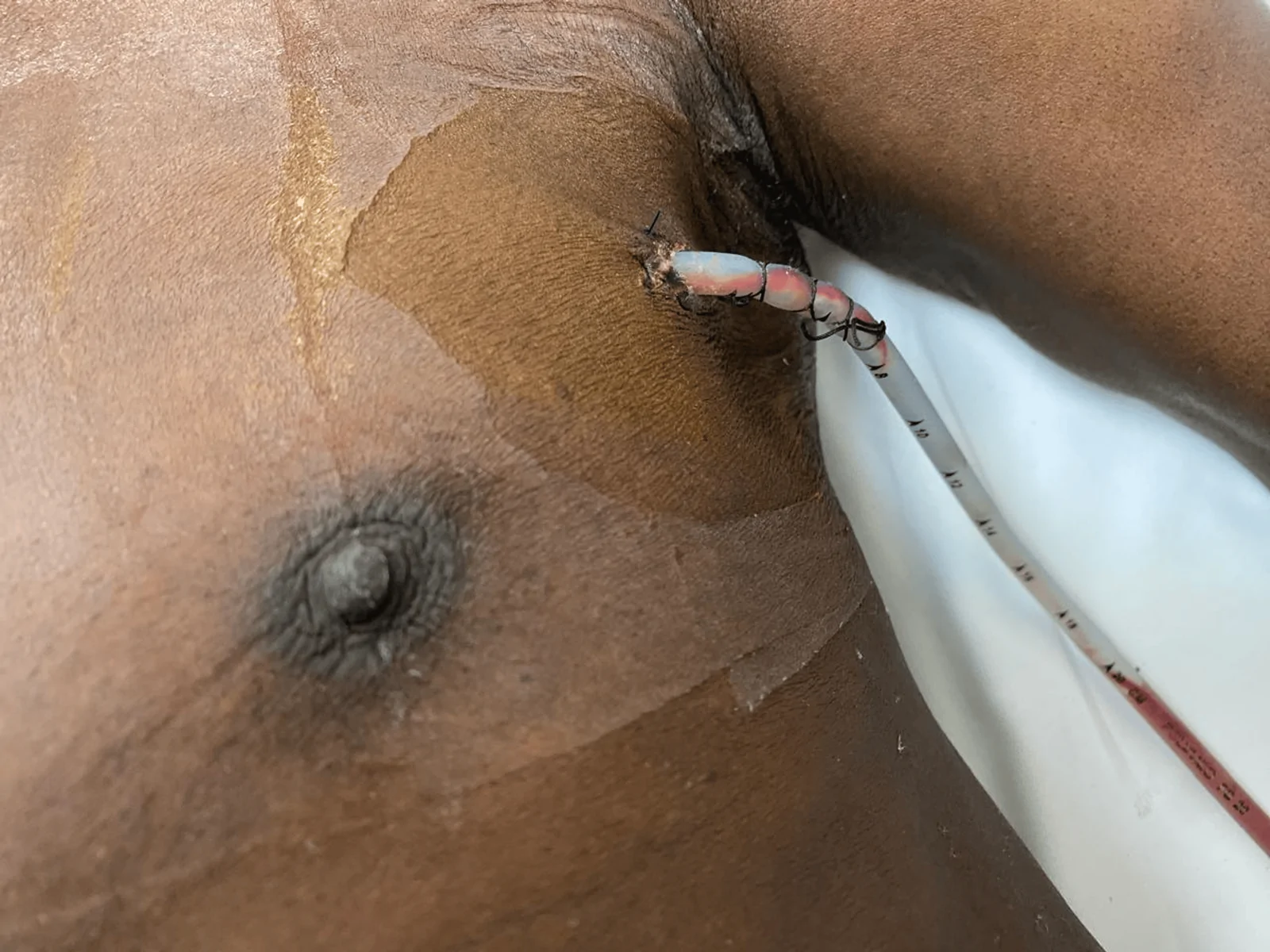
Left lateral chest wall showing a drain secured at the postoperative wound site

Despite initial improvement, the patient remained febrile with chest pain and fluctuance at the surgical sites. Laboratory studies revealed elevated inflammatory markers (CRP 443 mg/dL, procalcitonin 5.114 ng/mL, WBC 15,600/mm³). Due to these findings, intravenous clindamycin 600 mg was added as per infectious disease specialist opinion. Reexploration surgery was performed on December 19, 2024, which revealed recurrent abscesses and extensive necrosis, requiring repeat drainage and partial excision of the right pectoralis muscle. Multiple chest wall drains were inserted following debridement, with postoperative wound appearance and drain placement shown in Figures 4-6.

Postoperatively, the patient received intensive care, chest physiotherapy, and strict glycemic control with insulin. No intraoperative or postoperative complications were reported during or after the procedures. Clinical status improved, with resolution of fever and pain. He was discharged on December 25, as he appeared well with less serous fluid being drained from the wounds. The drains were removed as CRP and WBC on the day of discharge were 13.6 mg/L and 7,400 cells/mm^3^. He was discharged on oral meropenem 600 mg q12hrs and ciprofloxacin 500 mg q12hrs for one week along with insulin therapy, with instructions for follow-up.

Follow-up

At the two-week postoperative follow-up, the patient was clinically stable and reported no fever, chills, chest pain, lower limb pain, or other systemic symptoms suggestive of persistent infection. Chest wall examination showed healthy, well-formed granulation tissue with progressive wound healing. There was no surrounding erythema, swelling, tenderness, purulent discharge, wound dehiscence, or evidence of recurrent fluid collection. The left ankle wound had healed completely, without residual redness, swelling, or discharge, and the patient was able to bear weight and ambulate independently without discomfort.

At the one-month postoperative follow-up, the patient remained asymptomatic and continued to show satisfactory recovery. Both the chest wall and left ankle wounds had healed completely, with no clinical evidence of recurrent infection, new collections, or postoperative complications. He had resumed his usual daily activities, including walking without difficulty, and no further surgical or antimicrobial intervention was required.

At the three-month postoperative follow-up, the patient remained asymptomatic, with sustained healing of the chest wall and left ankle wounds and no clinical evidence of recurrent infection. He had preserved shoulder and lower limb function and had resumed his usual daily activities without limitation.

## Discussion

NF is a rare yet one of the deadliest complications of long-standing, untreated mucosal infections. It is an uncommon but rapidly progressive soft-tissue infection with significant morbidity and mortality. Globally, its incidence is estimated at 0.4-0.53 cases per 100,000 population annually, though regional variations exist depending on underlying risk factors and healthcare access. NF can affect individuals of any age, but it is more frequently observed in middle-aged and elderly patients, particularly those with comorbidities such as diabetes mellitus, obesity, chronic kidney disease, or immunosuppression. In diabetic individuals, impaired immune response and poor wound healing significantly increase the risk of developing NF. In our case, the patient had diabetes mellitus, which aligns with existing literature identifying diabetes as one of the most significant predisposing risk factors for NF [3].

NF is classified into four microbiological types. Type I, the most common, is polymicrobial and involves mixed aerobic and anaerobic organisms, including *E. coli*, Bacteroides, and Streptococcus species; it occurs more often in older or immunocompromised patients and in those with diabetes or peripheral vascular disease. Type II is usually monomicrobial, most commonly caused by group A Streptococcus, with or without *S. aureus*, and may rapidly progress to toxic shock syndrome even in otherwise healthy individuals. Type III is associated with marine organisms such as *Vibrio vulnificus*, typically following seawater exposure or ingestion of raw seafood. Type IV is fungal and is usually seen in severely ill or immunocompromised patients, commonly involving Candida or Zygomycetes species [5].

NF progresses through thrombosis of the small vessels supplying the fascia and subcutaneous tissues, resulting in tissue ischemia, liquefaction, and progressive necrosis. The overlying skin may initially appear relatively preserved despite extensive deep-tissue involvement, contributing to delayed recognition. During surgery, the presence of devitalized fascia, poor tissue resistance, and necrotic fluid supports the diagnosis. In the present patient, extensive fascial and subcutaneous tissue necrosis was identified intraoperatively, confirming that the process extended beyond a localized abscess or isolated muscle injury.

The clinical features of NF can range from high-grade fever, chills, cutaneous erythema, and diffuse tenderness of the affected area to altered mental status with hypotension and shock. The symptoms can be very vague at the early course of the disease, but then NF has a rapid progression, and the patient can be very ill in a short period of time. Similarly, our patient initially presented with nonspecific symptoms that rapidly progressed to severe systemic illness, highlighting the typical but deceptive early course of NF described in previous studies [6].

In the United States, the incidence of NF is estimated at approximately four cases per million people annually. Common comorbidities associated with NF-related deaths in the United States include diabetes mellitus and obesity. ​ In contrast, specific epidemiological data on NF in the UAE are limited. However, the UAE has a high prevalence of diabetes, which is a known risk factor for NF. This suggests that the burden of NF in the UAE could be significant, although comprehensive studies are needed to determine the exact incidence and mortality rates [7].

NF is strongly associated with a range of predisposing risk factors that compromise immune function or disrupt skin integrity, facilitating bacterial invasion into deeper soft tissues. Among the most significant risk factors is diabetes mellitus, which is present in up to 60% of cases and contributes through impaired neutrophil function, peripheral vascular disease, and delayed wound healing. Immunosuppression, whether due to malignancy, chemotherapy, corticosteroid use, HIV/AIDS, or organ transplantation, also significantly elevates risk. Local trauma, even minor or blunt injuries, surgical wounds, or skin breaches from injections, ulcers, or insect bites, often act as initiating events. Understanding these risk factors is critical for early recognition and timely intervention, especially in high-risk populations [8].

Diagnosis of NF can be challenging because early findings, including pain, swelling, erythema, fever, and tachycardia, may be nonspecific. Laboratory abnormalities such as leukocytosis, elevated CRP, hyponatremia, hyperglycemia, and renal dysfunction may increase clinical suspicion. The LRINEC score incorporates CRP, WBC count, hemoglobin, sodium, creatinine, and glucose. In our patient, the admission LRINEC score was 6, placing him in the intermediate-risk category. Imaging may help define the extent of infection, with ultrasound identifying soft-tissue collections and CT demonstrating fascial involvement, fluid tracking, or gas. However, imaging and scoring systems should support rather than replace clinical judgment, and definitive diagnosis may require urgent surgical exploration [9,10].

Effective management of NF requires a prompt, aggressive, and multidisciplinary approach. Early and extensive surgical debridement is essential to remove all necrotic and infected tissue, often necessitating multiple procedures to achieve adequate source control. Broad-spectrum intravenous antibiotics must be initiated immediately, targeting gram-positive organisms, including MRSA, gram-negative rods, and anaerobes. Common empiric regimens include combinations such as vancomycin, meropenem, and clindamycin, which may be adjusted according to culture and sensitivity results. Clindamycin is particularly valued for its ability to inhibit bacterial toxin production. The management approach in our case, involving early aggressive surgical debridement combined with broad-spectrum antibiotics, is consistent with current recommended treatment strategies. The favorable clinical outcome observed in our patient further supports the importance of early intervention in reducing morbidity and mortality. Supportive care in an intensive care setting is critical to address hemodynamic instability, sepsis, fluid and electrolyte imbalances, pain control, and nutritional needs [11,12].

Wound care plays a pivotal role in promoting healing and functional recovery. Negative pressure wound therapy is increasingly utilized after debridement to enhance granulation tissue formation, reduce edema, improve perfusion, and facilitate delayed closure or grafting, showing improved outcomes compared to conventional dressings. Antibiotic therapy should be continued until clinical and microbiological control is achieved, often transitioning to oral agents for outpatient management, with close infectious disease consultation to ensure optimal dosing and duration [13].

NF can cause extensive tissue destruction and may rapidly progress to sepsis, septic shock, and multiorgan failure. Local complications include skin and muscle necrosis, limb loss, large postoperative tissue defects, and the need for repeated debridement or reconstructive surgery. In chest wall disease, infection may also extend into deeper thoracic structures. Despite appropriate treatment, NF remains associated with substantial morbidity and mortality, particularly when diagnosis or surgical source control is delayed [14]. The clinical features, distribution of disease, microbiological findings, management, and outcomes of comparable published cases are summarized in Table 2.

**Table 2:** Comparison of the present case with previously reported chest wall and multifocal noncontiguous necrotizing soft-tissue infections NF: necrotizing fasciitis; CT: computed tomography; MRSA: methicillin-resistant *Staphylococcus aureus*

Study	Age/sex and risk factors	Distribution or source	Organism(s)	Management and outcome
Petreanu et al. [1]	Adult patient; active pulmonary tuberculosis with a destroyed left lung and an immunocompromised clinical state	Chest wall NF developed after pleural drainage for pyopneumothorax and a bronchopleural fistula	*Streptococcus pyogenes* isolated from the wounds and pleural fluid; acid-fast bacilli were detected in sputum	Repeated debridement, negative-pressure wound therapy, bronchial closure, and subsequent pneumonectomy; survived
Belliraj et al. [15]	Seven patients (five men and two women); mean age 58 years; all had poorly controlled diabetes mellitus	Primary thoracic wall NF with deep chest wall collections demonstrated on CT	Not specified in the accessible abstract	Extensive necrosectomy involving skin and adjacent muscle; five recovered and two died from postoperative septic shock
Xu and Gounder [16]	70-year-old woman; type 2 diabetes, previous splenectomy, multiple previous laparotomies, and an immunocompromised state	Multifocal noncontiguous infection involving the entire left lower limb and right gluteal region	No organism isolated, probably because broad-spectrum antibiotics had been administered before sampling	Extensive left-thigh debridement; disease progressed rapidly, and the patient received palliative care because of severe illness and poor prognosis
Bender et al. [17]	The patient’s exact age and full comorbidity details should be confirmed from the complete case text before inclusion	Bilateral, noncontiguous NF of the lower extremities	Escherichia coli	Aggressive treatment was undertaken, but the outcome was fatal
Present case	54-year-old man; type 2 diabetes mellitus and previous left tibial fracture with internal fixation	Bilateral chest wall NF with CT-reported mediastinal extension and noncontiguous left lower limb involvement; recent blunt pectoralis muscle injury was reported	MRSA, *Escherichia coli*, and Bacteroides spp.	Multiple chest wall and lower limb debridements, drain placement, broad-spectrum antibiotics, and glycemic control; complete recovery

Overall, our case aligns with existing literature regarding risk factors, clinical presentation, and management of NF, while emphasizing the importance of early recognition and prompt multidisciplinary intervention to improve patient outcomes.

Limitations

This report has several limitations. As a single case report, its findings cannot be generalized to all patients with chest wall or multifocal NF. Although the formal contrast-enhanced CT report documented bilateral chest wall collections with mediastinal extension, the original CT images were unavailable for publication and could not be independently reassessed. Furthermore, a causal relationship between the reported blunt pectoralis muscle injury and the subsequent infection could not be established. The mechanism underlying the noncontiguous chest wall and left lower limb involvement also remains uncertain, as hematogenous dissemination could not be confirmed. Although the patient demonstrated sustained wound healing and no clinical recurrence at three months, longer follow-up would be required to assess late recurrence and long-term functional outcomes.

## Conclusions

NF of the chest wall is a rare but life-threatening condition, particularly in patients with diabetes or other predisposing comorbidities. This case is distinctive because of the bilateral chest wall infection with mediastinal extension and the rare, noncontiguous involvement of the contralateral left lower limb. The presence of infection at anatomically distant sites highlights the importance of a comprehensive whole-body examination in patients with diabetes presenting with sepsis, even when symptoms appear to arise from a single region. Clinicians should maintain a high index of suspicion when rapidly progressive pain, swelling, erythema, or systemic toxicity is present. Imaging may help define the extent of disease but should not delay urgent surgical exploration when necrotizing infection is strongly suspected. Multiple surgical debridements, broad-spectrum antimicrobial therapy, glycemic control, and coordinated multidisciplinary care resulted in complete wound healing, preserved shoulder and lower limb function, and no clinical evidence of recurrence at the three-month follow-up.
